# Development of a multidisciplinary competency framework for specialist nurses in outpatient dental sedation and anesthesia: a mixed-methods Delphi and AHP study

**DOI:** 10.3389/fmed.2026.1835046

**Published:** 2026-06-16

**Authors:** Kai Li, Yuxue Wu, Shunyi Li, Weiping Wang, Ming Yi, Lin Fan

**Affiliations:** 1 Department of Medical Affairs, The Affiliated Stomatological Hospital of Chongqing Medical University, Chongqing, China; 2 Department of Anesthesiology, The Affiliated Stomatological Hospital of Chongqing Medical University, Chongqing, China; 3 Chongqing Key Laboratory of Oral Diseases and Biomedical Sciences, Chongqing, China; 4 Chongqing Municipal Key Laboratory of Oral Biomedical Engineering of Higher Education, Chongqing, China; 5 Students’ Affairs Division, Chongqing Three Gorges Medical College, Chongqing, China

**Keywords:** competency framework, Delphi method, dental sedation, NORA, patient safety, specialist nurse

## Abstract

**Background:**

The rapid global expansion of “comfortable dental care” has significantly increased the demand for outpatient sedation. However, the unique challenges of Non-Operating Room Anesthesia (NORA), including shared airways and limited rescue resources, necessitate highly specialized nursing competencies. This study aims to develop and validate a comprehensive competency framework for specialist nurses in outpatient dental sedation and anesthesia to enhance perioperative safety.

**Methods:**

Guided by the Onion Competency Model, a preliminary framework was established through a structured literature review and Behavioral Event Interviews (BEIs). A two-round modified Delphi consultation was conducted between August and October 2025 with a multidisciplinary panel of 25 experts (anesthesiologists, dental surgeons, and nursing specialists) from nine provinces in China. The Analytic Hierarchy Process (AHP) was employed to determine the relative weights of the indicators, with consistency ratios calculated to ensure logical rigor.

**Results:**

The final framework comprises 4 primary, 11 secondary, and 40 tertiary indicators. The four primary domains and their respective weights are: Professional Skills (0.361), Professional Knowledge (0.315), Personal Traits (0.179), and Professional Accomplishment (0.151). The expert authority coefficient (Cr) reached 0.847, and Kendall’s W for the second round showed significant consensus (*p* < 0.001). Notably, “Risk Warning Management” (0.289) emerged as the most critical secondary competency. The overall AHP consistency ratio was 0.034, indicating high reliability.

**Conclusion:**

This study establishes a scientifically validated benchmark for the recruitment, training, and evaluation of dental sedation nurses. By prioritizing risk-prevention skills and situational awareness, this framework provides a strategic blueprint for improving patient safety in ambulatory anesthesia settings within the Chinese healthcare context, with potential for cross-cultural adaptation.

## 1 Introduction

In the contemporary era of “comfortable dental care,” the utilization of sedation and anesthesia for outpatient dental procedures has experienced an unprecedented global expansion to manage patient anxiety and improve procedural efficiency (1). Outpatient sedation not only effectively alleviates dental phobia and anxiety but also provides an optimal environment for complex dental surgeries (2, 3). Within this specialized clinical setting, specialist nurses are recognized as indispensable stakeholders in ensuring patient safety. Their role has evolved beyond traditional chair-side assistance to encompass comprehensive perioperative management, including pre-anesthetic screening, intraoperative hemodynamic monitoring, airway protection, and post-sedation discharge assessment (4–6). Despite the high reliance on nursing expertise, the safety of dental sedation in Non-Operating Room Anesthesia (NORA) settings remains a significant concern (7, 8). Outpatient dental clinics often present unique challenges, such as confined surgical spaces, rapid patient turnover, and limited immediate access to advanced life support compared to general hospitals (3, 9). Consequently, nurses must serve as the primary safety monitors, possessing sophisticated clinical judgment and crisis-response capabilities to detect and prevent adverse events like respiratory depression or cardiovascular instability (10–12).

However, a critical discrepancy exists between the increasing clinical complexity and the current state of nursing specialization. While specialist nurses’ roles often vary across different clinical settings, general sedation nursing guidelines are not standardized or tailored to the unique demands of the dental outpatient environment (6, 9, 13). Current nursing guidelines are largely extrapolated from general perioperative standards, which frequently overlook the high-stakes “human factors” and crisis-response capabilities essential for dental anesthesia. This absence of a validated model addresses a critical gap in clinical standards, hindering the development of evidence-based training and potentially compromising patient safety (14, 15).

To address these gaps, establishing a robust competency framework is essential to define the professional boundaries and core requirements of this emerging specialty (16). This study utilizes the Onion Competency Model, originally proposed by Boyatzis, which provides a hierarchical and integrated perspective for defining professional competence (17–19). This model is particularly effective for distinguishing between readily observable technical skills (the outer layers) and the deeper, more stable personal attributes (the inner layers) that drive clinical excellence. By categorizing indicators into Professional Knowledge, Professional Skills, Professional Ethics, and Personal Attributes, this approach transcends mere task-based checklists to represent a holistic “nursing persona” required for safe outpatient dental sedation.

Therefore, the primary objective of this study was to develop and validate a standardized competency framework for specialist nurses in outpatient dental sedation and anesthesia. Specifically, this mixed-methods study aimed to: (1) identify the core dimensions and specific indicators of professional competency required for this unique ambulatory setting; (2) determine the relative importance and weight of each indicator using the Analytic Hierarchy Process (AHP); and (3) establish a scientifically grounded reference for the recruitment, specialized training, and performance evaluation of dental sedation nurses, thereby enhancing patient safety and nursing care quality in NORA settings.

## 2 Materials and methods

### 2.1 Study design

A mixed-methods, sequential exploratory study was conducted to develop and validate the competency framework. A mixed-methods design was selected because the development of a competency framework for this emerging specialty required both qualitative exploration (to identify context-specific competency indicators through literature and expert experience) and quantitative validation (to achieve consensus and determine relative weights). Neither approach alone could achieve both objectives: a qualitative-only study would lack the statistical rigor needed for a standardized framework, while a purely quantitative approach would risk overlooking the tacit, experience-based competencies essential for this unique clinical setting (20). The study followed the Guidance on Conducting and Reporting Delphi Studies (CREDES) to ensure methodological rigor (21); the completed checklist is provided in Supplementary material 1.

### 2.2 Phase 1: framework development

The preliminary framework was derived from two sources:

Structured literature review: A structured search of PubMed, Web of Science, CNKI, and WanFang (inception to August 1, 2025) was conducted using the following search terms: “competency framework,” “nurse specialist,” “nurse,” “sedation OR anesthesia,” and “dental,” along with their Chinese equivalents. Inclusion criteria were studies addressing nursing competencies related to sedation, anesthesia, or dental care settings, published in English or Chinese in peer-reviewed journals. Editorials, commentaries, conference abstracts, and studies unrelated to clinical nursing practice were excluded. This search yielded 546 articles, from which 58 preliminary indicators were extracted.

Behavioral event interviews (BEIs): To complement the literature findings with practice-based insights, Behavioral Event Interviews (BEIs) were conducted. BEI is a competency-based interview technique originally developed by McClelland (22) that elicits detailed accounts of critical workplace incidents. Semi-structured interviews using the STAR technique (Situation, Task, Action, Result) were conducted with seven multidisciplinary experts, including anesthesiologists, nursing specialists, and dental surgeons with extensive experience in dental sedation. Each interview lasted approximately 15–20 min. Detailed field notes were recorded during each session. The interview data were analyzed using content analysis to extract key competency themes, which were then integrated with the literature review findings to form the preliminary indicator pool of 58 items.

### 2.3 Phase 2: modified Delphi consultation

A two-round modified Delphi process was executed with a purposive sample of 25 experts from nine Chinese provinces. The Delphi process was “modified” in the following ways: (1) the initial indicator list was derived from the Phase 1 structured literature review and BEI findings, rather than being generated *de novo* by the panelists in an open first round as in classical Delphi; (2) two rounds were conducted, which is consistent with current Delphi methodology guidelines when clear consensus criteria are met (30); and (3) predefined consensus criteria were established *a priori*. Between rounds, experts received a summary of aggregated group scores and anonymized qualitative feedback to inform their re-evaluation. Expert Inclusion Criteria required minimum of a bachelor’s degree, senior professional title, and ≥10 years of experience in anesthesiology, dentistry, or nursing management. Experts evaluated the clinical importance of each indicator using a five-point Likert scale (ranging from 1 = “not important at all” to 5 = “very important”), with open-ended sections provided for qualitative, anonymous feedback. Following each round, the research group meticulously analyzed the quantitative scores and qualitative recommendations to refine the indicators for the subsequent round. The criteria for retaining an indicator included a mean importance score of 4.0, a minimum of 75% of experts rating the item as 4 or 5, and a coefficient of variation < 0.2, indicating a high level of group consensus.

### 2.4 Data collection

The entire study was conducted between August and October 2025. In Phase 1, BEI interviews were conducted in person, with detailed field notes recorded by the research team. In Phase 2, Delphi questionnaires were distributed electronically via email and WeChat, with each round allowing a two-week response window. Quantitative data (five-point Likert-scale ratings) and qualitative data (open-ended written comments) were collected concurrently within the same questionnaire instrument. The qualitative data from both BEI field notes and open-ended Delphi feedback were analyzed using content analysis. Two members of the research team independently reviewed the qualitative responses, identified recurring themes and suggestions, and categorized them according to the existing indicator framework. Discrepancies were resolved through discussion. The qualitative findings informed indicator refinement between Delphi rounds (e.g., rewording of items, merging of overlapping indicators, and addition of new items suggested by experts).

### 2.5 Statistical analysis

Statistical analyses were performed using SPSS version 25.0 and Yaahp 10.3 software. Descriptive statistics, including frequencies, proportions, means, and standard deviations, were calculated to summarize expert demographics and indicator scores. The reliability of the expert panel was assessed via the response rate, while the degree of expert authority (Cr) was calculated as the arithmetic mean of the judgment coefficient (Ca) and familiarity coefficient (Cs). The stability of expert opinions across the two rounds was measured using the coefficient of variation (CV), and the overall degree of coordination was evaluated using Kendall’s coefficient of concordance (Kendall’s W).

**Figure 1:**
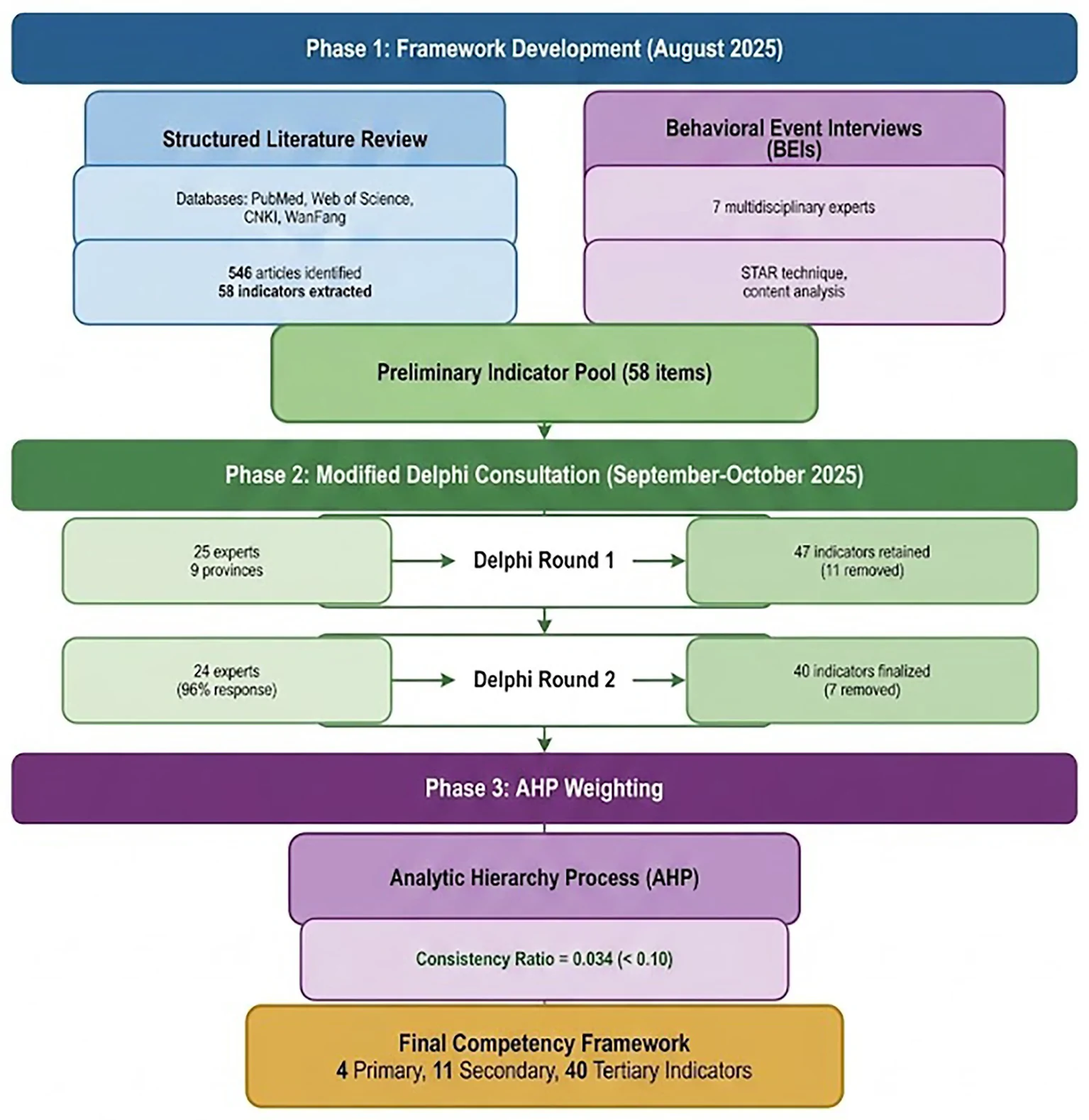
Study flow diagram.

Finally, the Analytic Hierarchy Process was utilized to determine the relative weights of the primary, secondary, and tertiary indicators. Crucially, the consistency ratio was calculated for each expert judgment matrix to verify the logical consistency of the weighting system, with a consistency ratio threshold of <0.10 deemed statistically acceptable (see Figure 1).

## 3 Results

### 3.1 Characteristics of the multidisciplinary expert panel

A geographically diverse, multidisciplinary panel of 25 experts from nine provinces and municipalities across China participated in the Delphi study. To ensure the framework addressed the complex, cross-disciplinary nature of NORA, the panel intentionally comprised 13 nursing specialists (52.0%), 9 anesthesiologists (36.0%), and 3 dental surgeons (12.0%). The participating experts demonstrated profound clinical experience, with an average age of 43.25 (standard deviation 8.15) years and an average professional tenure of 18.78 (standard deviation 7.86) years in their respective fields. Detailed demographic and professional characteristics are summarized in Table 1.

**Table 1:** Socio-demographic and professional characteristics of the multidisciplinary expert panel.

Variable	Round = 1 (*n* = 25) *n* (%)	Round = 1 (*n* = 24) *n* (%)
Gender		
Male	9 (36.0)	9 (37.5)
Female	16 (64.0)	15 (62.5)
Age(years)		
30 ~ 40	12 (48.0)	12 (50.0)
41 ~ 50	11 (44.0)	10 (41.67)
≥51	2 (8.00)	2 (8.33)
Current level of education		
Bachelors	11 (44.0)	11 (45.83)
Masters	10 (40.0)	9 (37.5)
Doctoral	4 (16.0)	4 (16.67)
Title		
Intermediate	19 (76.0)	18 (75.0)
Senior	6 (24.0)	6 (25.0)
Working experience (years)		
10 ~ 15	11 (44.0)	11 (45.83)
15–20	10 (40.0)	9 (37.5)
≥21	4 (16.0)	4 (16.67)
Professional status		
Anesthesiologist	9 (36.0)	9 (37.5)
Nurse	13 (52.0)	12 (50.0)
Dentist	3 (12.0)	3 (12.5)
Participation in sedation or anesthesia for dental treatment (years)		
≤5	8 (32.0)	7 (29.17)
6 ~ 10	12 (48.0)	12 (50)
≥11	5 (20.0)	5 (20.83)

### 3.2 Panel engagement and expert authority

The high level of panel engagement and expert authority strongly validated the reliability of the Delphi consultations. The response rates for the two iterative rounds were 100% (*n* = 25) and 96% (*n* = 24), respectively, indicating robust motivation and sustained participation among the experts.

The scientific authority of the panel was measured using the expert authority coefficient (Cr). The judgment coefficients (Ca) for the two rounds were 0.876 and 0.874, while the familiarity coefficients (Cs) were 0.811 and 0.819, respectively. Consequently, the calculated Cr values reached 0.844 and 0.847, substantially exceeding the widely accepted statistical threshold of 0.70 and confirming the high reliability of the experts’ evaluations. Furthermore, Kendall’s coefficient of concordance in the second round for primary, secondary, and tertiary indicators were 0.479, 0.256, and 0.286, respectively (*p* < 0.001), demonstrating a statistically significant consensus across the multidisciplinary panel (see Table 2).

**Table 2:** The competency framework for specialist nurses in outpatient dental sedation and anesthesia with corresponding weights derived from the analytic hierarchy process.

Delphi rounds	Items	Index (*n*)	Kendall’s W	*Χ* ^2^	*p*-value
Round 1	Primary indicators	4	0.468	36.184	**<**0.001
	Secondary indicators	11	0.202	55.854	**<**0.001
	Tertiary indicators	47	0.266	287.206	**<**0.001
Round 2	Primary indicators	4	0.479	33.624	**<**0.001
	Secondary indicators	11	0.256	60.147	**<**0.001
	Tertiary indicators	40	0.286	396.216	**<**0.001

### 3.3 The competency framework and indicator weights

Following the two-round Delphi consensus, the final standardized competency framework for specialist nurses in outpatient dental sedation and anesthesia was established, comprising 4 primary indicators, 11 secondary indicators, and 40 tertiary indicators.

The AHP was applied to calculate the relative importance of each domain. Crucially, the overall consistency ratio for the expert judgment matrix was 0.034. This value is significantly below the strict 0.10 threshold, verifying the robust logical consistency of the calculated weighting system.

The AHP analysis revealed the following relative weights for the four primary domains: Professional Skills (0.361), Professional Knowledge (0.315), Personal Traits (0.179), and Professional Accomplishment (0.151). Within the secondary indicators, “Risk Warning Management” was assigned the highest weight (0.289), identifying it as the most critical competency in the NORA setting, followed closely by “Relevant Knowledge of Sedation and Anesthesia” (0.244). The comprehensive indicator system and its corresponding weights are detailed in Table 3.

**Table 3:** Indicators and weights of the competency framework for dental sedation nurses.

Items of competency framework	Mean ± SD	Coefficient of variation	Weighting targets	Combination weights
1. Professional knowledge	4.67 ± 0.34	0.10	0.315	-
1.1 Relevant knowledge of stomatology	4.63 ± 0.43	0.15	0.114	0.013
1.1.1 Anatomic, physiological and pathological knowledge of oral cavity	4.55 ± 0.21	0.13	0.121	0.012
1.1.2 Treatment guideline of common oral diseases	4.63 ± 0.31	0.09	0.134	0.028
1.2 Relevant knowledge of oral care	4.43 ± 0.14	0.13	0.063	0.005
1.2.1 Framework of involvement in oral health promotion	4.21 ± 0.24	0.11	0.080	0.017
1.2.2 Knowledge of oral self-care	4.36 ± 0.13	0.17	0.091	0.011
1.3 Relevant knowledge of sedation and anesthesia	4.65 ± 0.49	0.13	0.244	0.018
1.3.1 Airway, respiratory, cardiovascular physiology and pathophysiology	4.67 ± 0.12	0.18	0.134	0.013
1.3.2 Function and evaluation of intermittent monitoring of cardiac rhythm, oxygenation and ventilation	4.51 ± 0.34	0.12	0.173	0.017
1.3.3 Pharmacological knowledge of sedation and anesthesia	4.49 ± 0.18	0.14	0.166	0.009
1.3.4 Principles of pre sedation or pre anesthesia evaluation and risk assessment	4.56 ± 0.21	0.11	0.094	0.003
1.3.5 Principles of report and track adverse effects of sedation and anesthesia	4.61 ± 0.24	0.12	0.093	0.008
1.3.6 Framework of pain management.	4.36 ± 0.25	0.13	0.168	0.024
1.3.7 Criteria of post-sedation or post anesthesia discharge	4.42 ± 0.18	0.11	0.126	0.007
1.4 Related knowledge of professional practice	4.42 ± 0.91	0.15	0.043	0.004
1.4.1 Principles of behavioral management and psychological intervention	4.26 ± 0.16	0.17	0.064	0.003
1.4.2 Principles of ongoing quality improvement audit in accordance with laws and ethics	4.12 ± 0.32	0.15	0.089	0.008
2. Professional skills	4.87 ± 0.36	0.12	0.361	-
2.1 Risk warning management	4.96 ± 0.21	0.04	0.289	0.109
2.1.1 Fall risk awareness and fall prevention during sedation and anesthesia	4.91 ± 0.23	0.07	0.285	0.015
2.1.2 Predict the risk of sedation and anesthesia	4.51 ± 0.16	0.14	0.162	0.009
2.1.3 Recognize and manage patient condition of over sedated or inadequately sedated	4.89 ± 0.29	0.04	0.131	0.051
2.2 Emergency response	4.92 ± 0.26	0.06	0.177	0.019
2.2.1 Proficient in basic first aid skills	4.73 ± 0.34	0.08	0.124	0.035
2.2.2 Evaluate and response of complication of sedation and anesthesia	4.96 ± 0.03	0.04	0.153	0.037
2.3 Nursing skills of sedation and anesthesia	4.83 ± 0.31	0.08	0.112	0.012
2.3.1 Proficient in the practice of four-handed dentistry	4.48 ± 0.46	0.15	0.118	0.033
2.3.2 Perform pre assessment of airway, jaw and fasting status	4.67 ± 0.24	0.13	0.166	0.041
2.3.3 Monitor airway and cardiovascular stability, recognize and feedback abnormal patterns	4.52 ± 0.21	0.15	0.172	0.053
2.3.4 Master the principles and safe application of anesthesia monitoring instruments and equipment	4.67 ± 0.36	0.13	0.210	0.011
2.3.5 Recognize, assess and manage pain and anxiety	4.63 ± 0.54	0.11	0.075	0.008
2.3.6 Document outcomes of sedation and anesthesia	4.39 ± 0.45	0.13	0.026	0.003
2.3.7 Determine safe discharge from monitored recovery	4.75 ± 0.43	0.09	0.113	0.011
3. Professional accomplishment	4.62 ± 0.43	0.13	0.151	-
3.1 Professional collaboration	4.74 ± 0.52	0.11	0.246	0.051
3.1.1 Lead practice closely related in cooperation with other professions	4.81 ± 0.49	0.11	0.190	0.023
3.1.2 Patient safety and nursing care	4.41 ± 0.54	0.13	0.091	0.018
3.1.3 Critical thinking skills	4.96 ± 0.21	0.03	0.087	0.017
3.1.4 Humanistic care	4.83 ± 0.29	0.07	0.113	0.012
3.1.5 Effective communication	4.51 ± 0.46	0.11	0.186	0.021
3.1.6 Self-efficacy	4.39 ± 0.25	0.16	0.191	0.039
3.2 Research and teaching ability	4.46 ± 0.17	0.13	0.068	0.003
3.2.1 Capacity to evidence-based approach in practice	4.91 ± 0.23	0.07	0.142	0.014
3.2.2 Academic teaching ability	4.83 ± 0.29	0.07	0.061	0.003
3.2.3 Scientific innovation ability	4.61 ± 0.36	0.11	0.098	0.005
4. Personal trait	4.42 ± 0.46	0.12	0.179	-
4.1 Personal characteristics	4.49 ± 0.21	0.12	0.098	0.005
4.1.1 Situational awareness	4.91 ± 0.36	0.12	0.142	0.034
4.1.2 Cautiousness	4.53 ± 0.43	0.08	0.136	0.007
4.1.3 Resilience	4.33 ± 0.39	0.13	0.128	0.015
4.1.4 Stress management	4.52 ± 0.68	0.15	0.039	0.004
4.2 Career development	4.36 ± 0.76	0.13	0.247	0.011
4.2.1 Professional ethics	4.31 ± 0.35	0.11	0.126	0.008
4.2.2 Organizational commitment	4.31 ± 0.49	0.14	0.160	0.021

## 4 Discussion

### 4.1 The criticality of risk warning management in NORA settings

Our AHP analysis revealed that “Professional Skills” (0.361) and its sub-dimension “Risk Warning Management” (0.289) carry the highest weights within the framework. This finding aligns with the unique safety profile of the Association of periOperative Registered Nurses (6) and the International Committee for the Advancement of Procedural Sedation (13). In outpatient dental clinics, the “shared airway” between the dentist and the anesthesia provider creates a high-risk environment for respiratory obstruction and hypoxia. Unlike traditional operating rooms, physical space is often constrained, and access to advanced life support may be delayed (23, 24). Therefore, a specialist nurse’s ability to proactively identify subtle signs of respiratory depression or hemodynamic instability is not merely a supplementary skill but a primary safety barrier (25, 26). This underscores the transition of the dental nursing role from a “technical assistant” to a “vigilant safety monitor.”

### 4.2 Integration of latent traits and clinical excellence via the onion model

**Figure 2:**
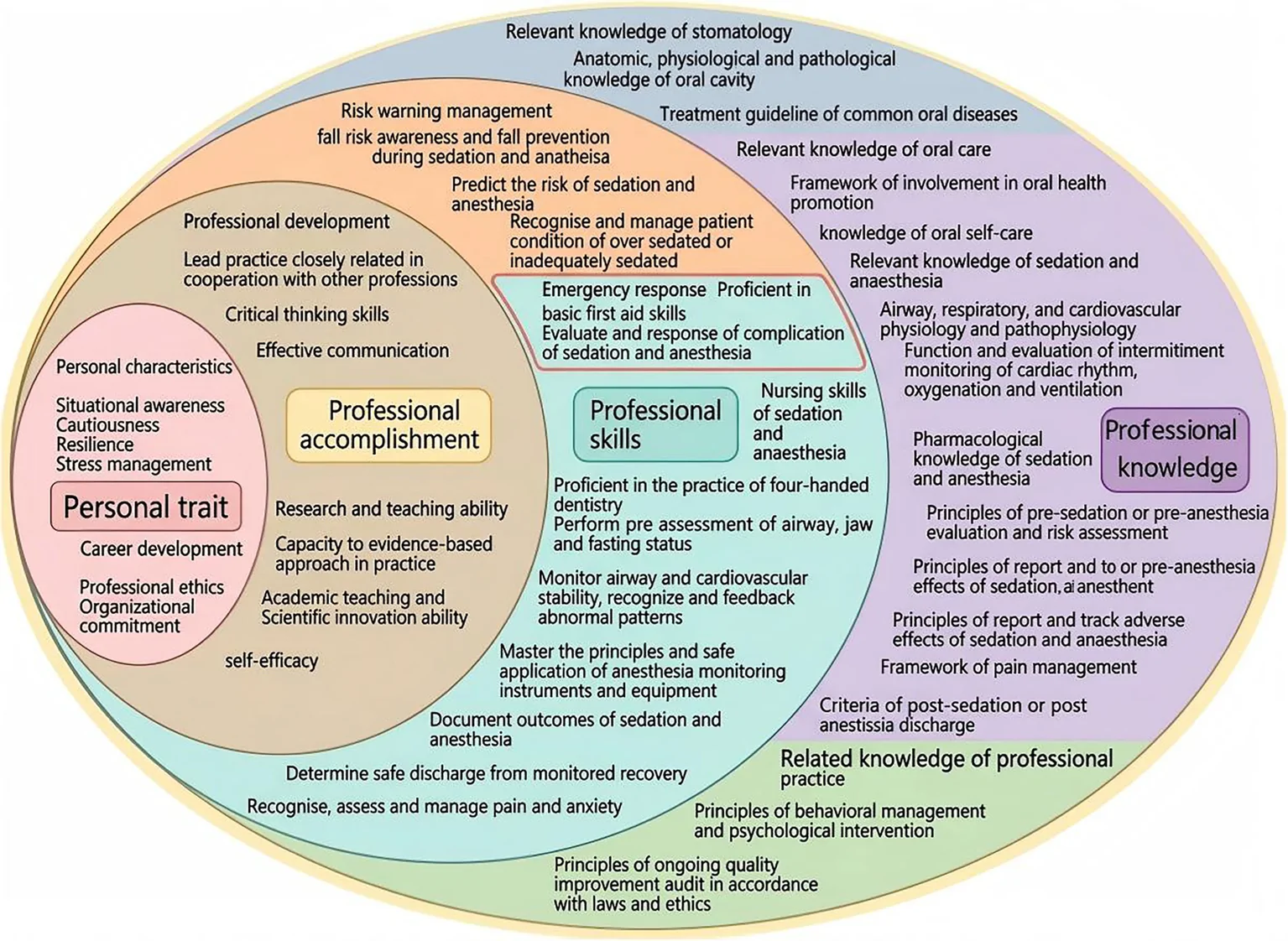
The conceptual framework based on the Onion Competency Model.

By employing the Onion Competency Model (Figure 2), this study identifies that “Personal Traits” (0.179)—such as situational awareness, decisiveness, and emotional stability—are foundational to professional excellence. While “Professional Knowledge” (0.315) forms the outer, observable layer of the onion, it is the inner core of psychological resilience and critical thinking that dictates performance during anesthesia crises. In dental sedation, where patient turnover is rapid and complications can escalate quickly, a nurse’s situational awareness allows for the “pre-emptive” management of adverse events. This holistic approach bridges the gap between theoretical knowledge and real-world clinical efficacy, advocating for atrecruitment and training strategy that values both “hard skills” and “soft attributes” (27).

### 4.3 Multidisciplinary collaboration as a safety determinant

The significant weight assigned to “Multidisciplinary Collaboration” (0.190) reflects the essential nature of the tripartite relationship between the anesthesiologist, the dentist, and the specialist nurse. Effective closed-loop communication is vital to prevent medication errors and manage the depth of sedation accurately (28). Our findings suggest that competency in dental sedation nursing is not an isolated set of tasks but is deeply embedded in the team’s collective ability to maintain patient safety. Establishing this framework provides a common language for MDT (Multidisciplinary Team) evaluations, ensuring that all providers operate under a unified standard of care (29).

### 4.4 Limitations and future directions

Despite the rigorous methodology and high expert consensus, several limitations should be acknowledged. First, the Delphi panel consisted of experts from nine provinces in China; thus, findings may be influenced by specific national clinical standards and healthcare policies. Given that the professional scope for dental sedation nursing varies considerably across jurisdictions, international generalizability should be interpreted with caution. The inclusion criteria (e.g., Chinese professional titles, national degree requirements) are specific to the Chinese healthcare system. Cross-cultural validation studies involving international expert panels are warranted before applying this framework in other countries.

Second, while the AHP provided a logically consistent weighting system, these weights represent expert perceptions rather than direct clinical outcomes. The empirical relationship between high competency scores and improved patient safety metrics—such as a reduction in sedation-related adverse events—remains to be quantified in future studies. Finally, the next critical step is to translate these indicators into standardized clinical training programs and objective assessment tools to evaluate the long-term impact on the quality of patient care.

## 5 Conclusion

This study successfully developed and validated a comprehensive competency framework for specialist nurses in outpatient dental sedation and anesthesia. Through a rigorous two-round Delphi-AHP process, we established a scientifically grounded system consisting of 4 primary and 40 tertiary indicators. The high expert authority coefficient (0.847) and logical consistency ratio (0.034) validate the framework’s reliability for clinical application.

By emphasizing the synergy between clinical expertise and latent psychological traits, this model transcends traditional task-oriented nursing. Ultimately, this framework serves as a standardized benchmark for recruitment, curriculum development, and performance assessment, providing a strategic blueprint for enhancing the safety and quality of “comfortable dental care” within the Chinese healthcare context. Future cross-cultural validation studies are recommended to facilitate international adaptation of this framework.

## Data Availability

The original contributions presented in the study are included in the article/Supplementary material, further inquiries can be directed to the corresponding author.

## References

[1] Mawhinney RL Hope A. Sedation for dental procedures. Anaesth Intensive Care Med. (2023) 24:431–4. doi: 10.1016/j.mpaic.2023.05.012

[2] Ogawa M Sago T Furukawa H. The reliability and validity of the Japanese version of the modified dental anxiety scale among dental outpatients. Sci World J. (2020) 2020:1–6. doi: 10.1155/2020/8734946, 32410911 PMC7211259

[3] Wiemer SJ Nathan JM Heggestad BT Fillmore WJ Viozzi CF Van Ess JM . Safety of outpatient procedural sedation administered by Oral and maxillofacial surgeons: the Mayo Clinic experience in 17,634 sedations (2004 to 2019). J Oral Maxillofac Surg. (2021) 79:990–9. doi: 10.1016/j.joms.2020.12.002, 33382992

[4] Cpnp-Pc PDD. A pediatric nurse practitioner–led moderate sedation service: our 7-year experience. J Nurse Pract. (2020) 16:366–70. doi: 10.1016/j.nurpra.2020.02.014

[5] Werthman JA Maxwell CA Dietrich MS Jordan LM Minnick AF. Moderate sedation education for nurses in interventional radiology to promote patient safety: results of a national survey. J Radiol Nurs. (2020) 39:200–6. doi: 10.1016/j.jradnu.2020.04.001

[6] Williams K. Guidelines in practice: moderate sedation and analgesia. AORN J. (2022) 115:553–64. doi: 10.1002/aorn.13690, 35616460

[7] Hara T Ozawa A Shibutani K Tsujino K Miyauchi Y Kawano T . Practical guide for safe sedation. J Anesth. (2023) 37:340–56. doi: 10.1007/s00540-023-03177-5, 36912977

[8] Montarroyos SS Payson A De La Vega C Pulido A. Outpatient sedation and risks (including dental). Pediatr Rev. (2023) 44:203–12. doi: 10.1542/pir.2022-005642, 37002353

[9] Guttormson JL Chlan L Tracy MF Hetland B Mandrekar J. Nurses' attitudes and practices related to sedation: a National Survey. Am J Crit Care. (2019) 28:255–63. doi: 10.4037/ajcc2019526, 31263007 PMC6760853

[10] Hadfield A Thompson S Hall J Diaz-Navarro C. Perception of simulation training in emergencies for dental sedation practitioners. Clin Teach. (2018) 15:52–6. doi: 10.1111/tct.12626, 28296158

[11] Miles E Shah A. An audit of dental nurses' confidence with intravenous sedation within an oral surgery department at a UK dental hospital. Prim Dent J. (2022) 11:104–7. doi: 10.1177/20501684221112509, 36073054

[12] Radford PJ McKay A. The self-perceived role of sedation-trained dental nurses within a community dental service: a qualitative study. Br Dent J. (2020) 229:1–5. doi: 10.1038/s41415-020-1927-6, 32801326

[13] Leroy PL Krauss BS Costa LR Barbi E Irwin MG Carlson DW . Procedural sedation competencies: a review and multidisciplinary international consensus statement on knowledge, skills, training, and credentialing. Br J Anaesth. (2025) 134:817–29. doi: 10.1016/j.bja.2024.07.036, 39327154 PMC11867087

[14] Almarwani AM Alzahrani NS. Factors affecting the development of clinical nurses' competency: a systematic review. Nurse Educ Pract. (2023) 73:103826. doi: 10.1016/j.nepr.2023.103826, 37951064

[15] Dugan MA Altmiller G. AACN essentials and nurse practitioner education: competency-based case studies grounded in authentic practice. J Prof Nurs. (2023) 46:59–64. doi: 10.1016/j.profnurs.2023.02.003, 37188423

[16] Franks-Meeks S. Clinical staff nurse leadership: identifying gaps in competency development. Nurs Forum. (2018) 53:35–9. doi: 10.1111/nuf.12217, 28745441

[17] Marrelli AF Tondora J Hoge MA. Strategies for developing competency models. Admin Pol Ment Health. (2005) 32:533–61. doi: 10.1007/s10488-005-3264-0, 16082796

[18] Niu A Ma H Chen Z Zhang S Deng J Luo Y. Exploring the competencies of Chinese critical care nurses in mobile medical teams based on the onion model: a qualitative study. Nurs Crit Care. (2024) 29:868–79. doi: 10.1111/nicc.12981, 37743055

[19] Jiang X Ding Z Wang F Wang Z Wang W Xing Y . Construction of a competency framework of dental hygienists: a Delphi study. Nurse Educ Pract. (2023) 70:103692. doi: 10.1016/j.nepr.2023.103692, 37379696

[20] Creswell JW Plano Clark VL. Designing and Conducting Mixed Methods Research. 3rd ed. Los Angeles, CA: Sage (2018).

[21] Jünger S Payne SA Brine J Radbruch L Brearley SG. Guidance on conducting and REporting DElphi studies (CREDES) in palliative care: recommendations based on a methodological systematic review. Palliat Med. (2017) 31:684–706. doi: 10.1177/0269216317690685, 28190381

[22] McClelland DC. Identifying competencies with behavioral-event interviews. Psychol Sci. (1998) 9:331–3. doi: 10.1111/1467-9280.00065

[23] Hoffmann B Erwood K Ncomanzi S Fischer V O'Brien D Lee A. Management strategies for adult patients with dental anxiety in the dental clinic: a systematic review. Aust Dent J. (2022) 67 Suppl 1:S3–S13. doi: 10.1111/adj.12924, 35735746 PMC9796536

[24] Zhang J Zeng J Zhou P Deng H Yu C. Bibliometric analysis of pediatric dental sedation research from 1993 to 2022. Heliyon. (2024) 10:e25527. doi: 10.1016/j.heliyon.2024.e25527 38333804 PMC10850579

[25] Werthman JA Maxwell CA Dietrich MS Jordan LM Minnick AF. Moderate sedation education for nurses in interventional radiology to promote patient safety: results of a national survey. J Radiol Nurs. (2021) 40:49–55. doi: 10.1016/j.jradnu.2020.10.007

[26] Crego N. Procedural sedation practice: a review of current nursing standards. J Nurs Regul. (2015) 6:50–6. doi: 10.1016/S2155-8256(15)30036-7, 35295939 PMC8923528

[27] Kalladka M Markman S Raman KR Mansdorf A. Psychological factors determining prognosis of dental treatments. Dent Clin N Am. (2024) 68:739–50. doi: 10.1016/j.cden.2024.05.005 39244254

[28] Burtea D Dimitriu A Maloș A Cherciu I Săftoiu A. Assessment of the quality of outpatient endoscopic procedures by using a patient satisfaction questionnaire. Curr Health Sci J. (2019) 45:316–20. doi: 10.12865/CHSJ.45.03.11, 31297263 PMC6592669

[29] Pero-Cleveland E Rabey J Mosher A Geraci E DePaul S Hutchinson D . Benefit of an interdisciplinary pediatric sedation team with a clinical pharmacist on outpatient appointment duration and procedural sedation time. Paediatr Anaesth. (2026) 36:81–7. doi: 10.1111/pan.70069, 41118132

[30] Hasson F Keeney S McKenna H. Revisiting the Delphi technique - Research thinking and practice: a discussion paper. Int J Nurs Stud. (2025) 168:105119. doi: 10.1016/j.ijnurstu.2025.105119, 40383005

