# Correction: Development of a multidisciplinary competency framework for specialist nurses in outpatient dental sedation and anesthesia: a mixed-methods Delphi and AHP study

**DOI:** 10.3389/fmed.2026.1945912

**Published:** 2026-08-19

**Authors:** Kai Li, Yuxue Wu, Shunyi Li, Weiping Wang, Ming Yi, Lin Fan

**Affiliations:** 1 Department of Medical Affairs, The Affiliated Stomatological Hospital of Chongqing Medical University, Chongqing, China; 2 Chongqing Key Laboratory of Oral Diseases and Biomedical Sciences, Chongqing, China; 3 Chongqing Municipal Key Laboratory of Oral Biomedical Engineering of Higher Education, Chongqing, China; 4 Department of Anesthesiology, The Affiliated Stomatological Hospital of Chongqing Medical University, Chongqing, China; 5 Students' Affairs Division, Chongqing Three Gorges Medical College, Chongqing, China

**Keywords:** competency framework, Delphi method, dental sedation, NORA, patient safety, specialist nurse

Author Lin Fan was erroneously omitted as corresponding author. The correct corresponding authors are Ming Yi and Lin Fan.

The **Author contributions** Statement has been corrected to read:

KL: Methodology, Writing – original draft. YW: Formal analysis, Writing – review & editing. SL: Conceptualization, Data curation, Writing – review & editing. WW: Investigation, Project administration, Resources, Writing – original draft. MY: Writing – review & editing. LF: Supervision, Writing – review & editing

The order of authors in the author list and affiliations of the published paper was erroneously given as:

Kai Li^1, 2, 3^, Yuxue Wu^2, 3, 4^, Shunyi Li^2, 3, 4^, Weiping Wang^2, 3, 4^, Lin Fan^2, 3, 4^ and Ming Yi^5*^

1.Department of Medical Affairs, Stomatological Hospital of Chongqing Medical University, Chongqing, China.

2.Department of Anesthesiology, Stomatological Hospital of Chongqing Medical University, Chongqing, China.

3.Chongqing Key Laboratory of Oral Diseases and Biomedical Sciences, Chongqing, China.

4.Chongqing Municipal Key Laboratory of Oral Biomedical Engineering of Higher Education, Chongqing, China.

5.Students' Affairs Division at Chongqing Three Gorges Medical College, Chongqing, China

The correct author list reads:

Kai Li^1, 3, 4^, Yuxue Wu^2, 3, 4^, Shunyi Li^2, 3, 4^, Weiping Wang^2, 3, 4^, Ming Yi^5^ and Lin Fan^2, 3, 4^

^1^Department of Medical Affairs, The Affiliated Stomatological Hospital of Chongqing Medical University, Chongqing, China

^2^Department of Anesthesiology, The Affiliated Stomatological Hospital of Chongqing Medical University, Chongqing, China

^3^Chongqing Key Laboratory of Oral Diseases and Biomedical Sciences, Chongqing, China

^4^Chongqing Municipal Key Laboratory of Oral Biomedical Engineering of Higher Education, Chongqing, China

^5^Students' Affairs Division, Chongqing Three Gorges Medical College, Chongqing, China

^*^Corresponding author: Ming Yi (20130323@cqtgmc.edu.cn); Lin Fan (500741@hospital.cqmu.edu.cn)

The original version of this article has been updated.

