# Supplementary material for: Development of a multidisciplinary competency framework for specialist nurses in outpatient dental sedation and anesthesia: a mixed-methods Delphi and AHP study
Source: Front Med (Lausanne). 2026 Jun 16;13:1835046. doi: 10.3389/fmed.2026.1835046 (PMC13314621; doi:10.3389/fmed.2026.1835046)
Supplement: Supplementary file 1 [file Table_1.docx]

**Supplementary Material 1: CREDES Checklist**

*Guidance on Conducting and REporting DElphi Studies (Jünger et al., 2017)*

**Study:** Development of a Multidisciplinary Competency Framework for Specialist Nurses in Outpatient Dental Sedation and Anesthesia: A Mixed-Methods Delphi and AHP Study

| **Item No.** | **CREDES Recommendation** | **Reported (Yes/No)** | **Location in Manuscript** |
| --- | --- | --- | --- |
| 1 | Purpose of the Delphi study | Yes | Section 1 (Introduction) |
| 2 | Justification for using a Delphi approach | Yes | Section 2.1 |
| 3 | Pilot testing of questionnaire | **No** | **Not reported** |
| 4 | Criteria for selection of experts | Yes | Section 2.3 |
| 5 | Number of experts invited and justification | Yes | Section 2.3, Table 1 |
| 6 | Characteristics of experts (professional background) | Yes | Section 3.1, Table 1 |
| 7 | Description of expert panel composition | Yes | Section 3.1, Table 1 |
| 8 | Description of information provided to panelists | Yes | Section 2.3 |
| 9 | Number of rounds and justification | Yes | Section 2.3 |
| 10 | Description of each Delphi round | Yes | Section 2.3 |
| 11 | Description of how results were fed back | Yes | Section 2.3 |
| 12 | Criteria for consensus (defined a priori) | Yes | Section 2.3 |
| 13 | Criteria for revising or dropping items | Yes | Section 2.3 |
| 14 | Procedure when consensus was not reached | Yes | Section 2.3 |
| 15 | Summary of results per round | Yes | Section 3.2, Table 2 |
| 16 | Final results including consensus levels | Yes | Section 3.3, Table 3 |
| 17 | Ethical approval | Yes | Ethical Considerations |
| 18 | Anonymity of responses | Yes | Section 2.3 |
| 19 | Attrition across rounds | Yes | Section 3.2 |
| 20 | Acknowledgment of limitations | Yes | Section 4.4 |

**Reference:** *Jünger S, Payne SA, Brine J, Radbruch L, Brearley SG. Guidance on Conducting and REporting DElphi Studies (CREDES) in palliative care: Recommendations based on a methodological systematic review. Palliat Med. 2017;31(8):684-706.*

**Summary: 19 of 20 CREDES items reported (95%). Item 3 (Pilot testing of questionnaire) was not reported.**
